# Noncompaction and Takotsubo Syndrome in a Neuromuscular Disorder

**DOI:** 10.1155/2019/6902751

**Published:** 2019-06-03

**Authors:** Josef Finsterer, Claudia Stöllberger, Walter Benedikt Winkler

**Affiliations:** ^1^Krankenanstalt Rudolfstiftung, Vienna, Austria; ^2^2nd Medical Department with Cardiology and Intensive Care Medicine, Krankenanstalt Rudolfstiftung, Vienna, Austria

## Abstract

**Background:**

Takotsubo syndrome (TTS) in patients with left ventricular hypertrabeculation/noncompaction (LVHT) has been reported in four patients, and a TTS plus LVHT plus a neuromuscular disorder (NMD) was only reported once so far. Here, we present the fifth patient with LVHT and TTS and the second patient with LVHT, TTS, and a NMD.

**Methods and Results:**

The patient is a 68 yo female hobby choir singer with a history of skin dermatofibroma, skin fibrokeratoma, arterial hypertension, hyperlipidemia, hypothyroidism, anemia, hyponatremia, diverticulosis, LVHT detected at age 60 y, five syncopes, a liver cyst, and carotid endarterectomy 2 months prior to admission because of sudden-onset chest pain. Workup revealed ST elevation, troponin elevation, and mild coronary artery sclerosis. Ventriculography and transthoracic echocardiography (TTE) showed the apical type of a TTS. ECG normalised within 10 w and TTE within 6 w under beta-blockers and ATII-blockers. The TTS was triggered by being offended of being unable to sing anymore after endarterectomy. Neurological workup suggested the presence of a NMD.

**Conclusions:**

This case shows that LVHT occurs in NMD patients and that patients with LVHT and a NMD may develop a TTS. Whether patients with LVHT and a NMD are particularly prone to develop a TTS requires further confirmation. NMD patients with LVHT should avoid stress not to trigger a TTS.

## 1. Introduction

Only few patients with left ventricular hypertrabeculation (LVHT), also known as noncompaction, have been reported who also developed a Takotsubo syndrome (TTS) [1–4]. This unusual combination has been reported in a patient with a Beals-Hecht syndrome [1], a female with myotonic dystrophy type 1 [2], a 76 yo female without other concomitant diseases [3], and in a 12 yo female with low-grade glioma of the cerebellar vermis [4]. Here, we report a fifth patient with LVHT who developed a TTS shortly after carotid endarterectomy.

## 2. Methods and Results

**Figure 1:**
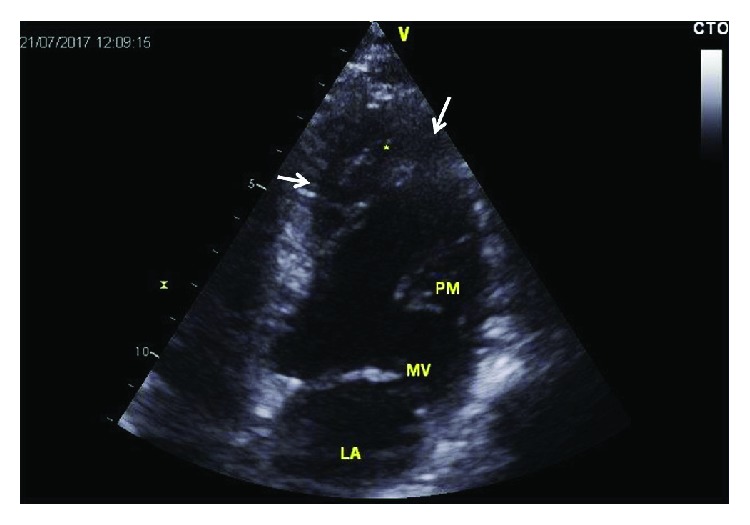
Echocardiographic apical five chamber view from 2017 showing hypertrabeculation/noncompaction in the apex (arrows). LA: left atrium; MV: mitral valve; PM: anterolateral papillary muscle.

The patient is a 68 yo Caucasian female, height 170 cm, weight 68 kg, with a previous history of smoking (20 pack-years) until age 50 y, arterial hypertension since age 40 y, and resection of a dermatofibroma on the right upper arm and a fibrokeratoma of the 3^rd^ digit of the left foot at age 50 y. The individual history was further positive for struma nodosa and Hashimoto thyroiditis since age 58 y, hyperlipidemia since at least age 58 y, a liver cyst in segments 6/7, diverticulosis of the colon, first detected at age 58 y, and myoma resection. Also, since age 58 y, she temporarily experienced a few panic attacks for which she was hospitalised once at age 60 y. Since age 59 y, she reported a mild sleep disorder. Mild anemia of unknown cause was recognised since age 59 y. Since at least age 59 y, recurrent hyponatremia in the absence of taking diuretics or serotonin reuptake inhibitors was noted. LVHT was first diagnosed at age 60 y and confirmed at age 68 y (Figure 1). Since age 63 y, she experienced recurrent nausea and abdominal pain with a tendency for constipation and flatulence. Gastroscopy and colonoscopy only revealed the known diverticulosis. Workup for secondary arterial hypertension at age 67 y was negative. Parathyroid hormone, renin, aldosterone, and ACTH levels were all in the normal range. There was recurrent transient lymphocytosis, but workup for malignancy was negative. The family history was positive for epilepsy (2^nd^ brother), dementia (2^nd^ brother), migraine (daughter), arterial hypertension (father, mother, and 1^st^ sister), arrhythmias (2^nd^ sister), diabetes (father), tuberculosis with osteomyelitis (1^st^ sister), Graves' disease (daughter), and coronary heart disease (father and 1^st^ brother). She was a passionate hobby choir singer since years.

Workup for two syncopes at age 67 y (the second with secessus urinae) and three syncopes before revealed normal systolic function and confirmed previously diagnosed LVHT on transthoracic echocardiography (TTE). Long-term electrocardiogram (ECG) had not been carried out so far. Concerning the neurological history, she reported muscle cramps under simvastatin, exercise intolerance, and easy fatigability. Upon the neurological exam, an exaggerated masseter reflex, generally reduced tendon reflexes, and fasciculations of the right adductor pollicis muscle were found. Electroencephalography (EEG), cerebral computed tomography (CCT), and cerebral magnetic resonance imaging (MRI) were normal, but carotid ultrasound and MR angiography (MRA) revealed a high-grade stenosis of the left internal carotid artery. This is why she underwent carotid endarterectomy of the left internal and common carotid artery. Postoperatively, she experienced recurrent collapses, hypertensive crises, and uncertainty and fear but no further syncope.

**Figure 2:**
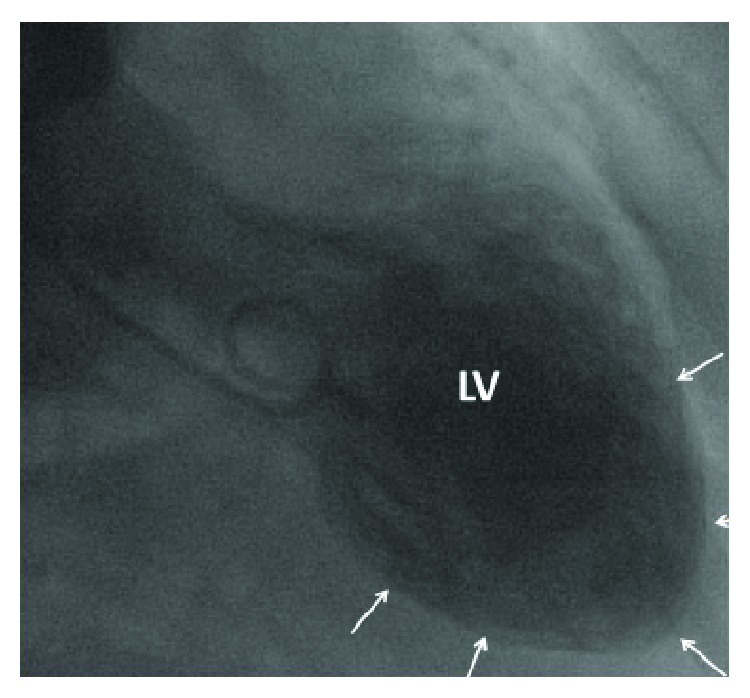
Ventriculography showing apical ballooning (arrows) of the left ventricle (LV).

Two months after surgery, she developed sudden onset thoracic pain. Since ECG showed ST elevation and troponin was elevated, myocardial infarction was suspected. However, acute coronary angiography revealed only mild coronary sclerosis but ventriculography showed the apical type of a TTS (Figure 2), which was confirmed by TTE. Already 6 d after the onset of symptoms, TTE showed almost normal systolic function, mild myocardial thickening, diastolic dysfunction grade 2, and regional apical akinesia/hypokinesia. TTE normalised completely within 6 w and the ECG within 10 w. The presumed trigger of the TTS was being offended of being unable to sing again. The cause of hyponatremia remained speculative. A syndrome of inadequate ADH secretion (SIADH) was excluded, and the patient was asked to salt the food more extensively. She is currently taking acetyl-salicylic acid, bisoprolol, candesartan, rilmenidine, and simvastatin.

## 3. Discussion

The patient is interesting for the coincidence of LVHT and a TTS and the presence of a NMD. A coincidence of LVHT and a TTS has been reported only in four patients before [1–4], suggesting that LVHT does not promote the development of a TTS. However, systematic investigations on the question if cardiomyopathy is generally a predisposing factor for the development of a TTS are not available. Thus, it is still conceivable that cardiomyopathy is less resistant to a catecholamine storm than the normal myocardium and that LVHT is nonetheless a risk factor for the development of a TTS.

Concerning the notion that LVHT occurs in up to 80% of the patients with a NMD [5], the present case confirms such a relation. The neurological history revealed muscle cramps under simvastatin, exercise intolerance, and easy fatigability, and the neurological exam revealed generally reduced tendon reflexes and fasciculation in the right adductor pollicis muscle, suggesting the presence of a NMD. The type of NMD, however, remained speculative since further workup for a NMD was not feasible. Anyhow, a metabolic NMD is more likely than a structural NMD.

LVHT is frequently complicated by ventricular arrhythmias, heart failure, or embolism [6]. None of these conditions had been recognised yet in the present patient, but it cannot be excluded that TTS is another, hitherto, not recognised complication of LVHT. In this respect, it is conceivable that already little distress, less than in cardiologically healthy patients, triggers the development of a TTS. Whether the five syncopes were rather due to ventricular arrhythmias, due to transient heart failure, or due to cardiovascular embolism than due to the carotid artery stenosis remains speculative. Seizures were excluded as a cause of the syncopes.

In conclusion, this case shows that LVHT occurs in NMD patients and that patients with LVHT and a NMD may experience a TTS. Whether patients with LVHT and a NMD are particularly prone to develop a TTS requires further investigations. NMD patients with LVHT should avoid extreme stress not to trigger a TTS.
